# Exploring physics students’ engagement with online instructional videos in an introductory mechanics course

**DOI:** 10.1103/PhysRevPhysEducRes.13.020138

**Published:** 2017-12-18

**Authors:** Shih-Yin Lin, John M. Aiken, Daniel T. Seaton, Scott S. Douglas, Edwin F. Greco, Brian D. Thoms, Michael F. Schatz

**Affiliations:** 1 Department of Physics, National Changhua University of Education, Changhua 500, Taiwan; 2 Centre for Computing in Science Education, Department of Physics, University of Oslo, 0316 Oslo, Norway; 3 VPAL Research, Harvard University, Cambridge, Massachusetts 02139, USA; 4 School of Physics, Georgia Institute of Technology, 830 State Street, Atlanta, Georgia 30332, USA; 5 Department of Physics and Astronomy, Georgia State University, Atlanta, Georgia 30303, USA

## Abstract

The advent of new educational technologies has stimulated interest in using online videos to deliver content in university courses. We examined student engagement with 78 online videos that we created and were incorporated into a one-semester flipped introductory mechanics course at the Georgia Institute of Technology. We found that students were more engaged with videos that supported laboratory activities than with videos that presented lecture content. In particular, the percentage of students accessing laboratory videos was consistently greater than 80% throughout the semester. On the other hand, the percentage of students accessing lecture videos dropped to less than 40% by the end of the term. Moreover, the fraction of students accessing the entirety of a video decreases when videos become longer in length, and this trend is more prominent for the lecture videos than the laboratory videos. The results suggest that students may access videos based on perceived value: students appear to consider the laboratory videos as essential for successfully completing the laboratories while they appear to consider the lecture videos as something more akin to supplemental material. In this study, we also found that there was little correlation between student engagement with the videos and their incoming background. There was also little correlation found between student engagement with the videos and their performance in the course. An examination of the in-video content suggests that students engaged more with concrete information that is explicitly required for assignment completion (e.g., actions required to complete laboratory work, or formulas or mathematical expressions needed to solve particular problems) and less with content that is considered more conceptual in nature. It was also found that students’ in-video accesses usually increased toward the embedded interaction points. However, students did not necessarily access the follow-up discussion of these interaction points. The results of the study suggest ways in which instructors may revise courses to better support student learning. For example, external intervention that helps students see the value of accessing videos may be required in order for this resource to be put to more effective use. In addition, students may benefit more from a clicker question that reiterates important concepts within the question itself, rather than a clicker question that leaves some important concepts to be addressed only in the discussion afterwards.

## I Introduction

With the advent of new online educational technologies, there is increasing interest in leveraging web-based resources in university courses. Using these resources, instructors are finding new ways to engage students both inside and outside of class [1-10]. For example, several institutions have incorporated online resources such as multimedia learning modules, computer simulation, e-Text as assignments that students should complete before coming to class in their introductory physics courses [6-10]. With an understanding of students’ engagement in these preparation activities and the content students still find difficult or confusing, instructors can then tailor the in-class lecture activities to engage students in higher levels of learning.

The use of web-based instructional tools has also invigorated interest in data-driven education [11-15], particularly due to the sheer size and scope of data being collected by platforms. Many of these platforms are able to provide second-by-second records of student engagement with resources in a course. Such data promise to provide educational researchers powerful and unprecedented insight into student learning behaviors [11-13]. For example, by analyzing how students engage online content, educators can begin to paint a picture of what resources students attend to and how they attend to these resources [11]. The effects of these online resources on student learning outcomes can also be explored.

In 2013, instructional videos that we originally created for a massively open online course (MOOC) were implemented in an introductory mechanics course for residential students at the Georgia Institute of Technology. The design of this course was inspired by the flipped classroom model [16], which suggests that the direct content delivery activities can be moved to an individual learning environment in order to save precious in-class time for activities involving more interactive engagement. In particular, in this course, the traditional in-class lectures were replaced by online videos that students were instructed to access outside of class at their convenience. The scheduled in-class periods, on the other hand, were used for group collaborative work. As part of an effort to investigate student participation and learning in this new type of physics course at Georgia Institute of Technology, we explored student engagement with these online instructional videos.

In our course, the videos were hosted on a Coursera platform that is open for registered Georgia Tech students only. This platform records not only whether a student accesses a video, but also every interaction a student makes with their video player in a tabulated time stamped output (e.g., pauses, plays, and seeks, which indicate when a student is skipping parts of the video to find a segment of interest). These records allow educators to investigate how students engage with videos in numerous ways. For the goal of our paper, we focus on the following three types of analysis, with relevant research questions presented below

(1) The extent to which students access videos. Fall 2013 was the first time in-class introductory mechanics lectures were replaced by online videos at the Georgia Institute of Technology. Therefore, understanding student use of this new online resource is of interest to instructors and researchers. Research questions in this realm include the following:
  - Did students click on the links to access these instructional videos?
  - If students decided to access a video, did they access the video completely, or did they access only parts (but not all) of the video? More specifically, for each video, what fraction of students accessed “almost” all of the video?
  - What videos (if any) did students access more?
  In this paper, we use the term starting a video-accessing session to refer to the action of a student clicking on the link for a video to access the content through online streaming or to download it. Note that if the same student clicks on the link for the same video multiple times, multiple video- accessing sessions are made. The number of unique videos accessed by a given student, on the other hand, refers to how many *different* videos links they had ever clicked on. It does not take into account how many times any given video is accessed by the same student. We also note that while the first question here focuses on whether students clicked on the video links (i.e., whether they started any accessing sessions with the given videos), the 2nd question looks deeper into the amount of content students interacted with after the video started playing.
(2) Relation between video accessing and student performance.
  With an understanding of how students in the course accessed videos, we then investigate if students’ video accessing behaviors (such as the number of videos accessed, the time spent watching video, or the interaction frequency with video player) is correlated to their incoming background and their performance in the course.
(3) Detailed student interaction with videos.
  In order to help understand the relation between video accessing and student performance further, an exploration into students’ detailed interaction with videos is discussed. In particular, we investigate the types of in-video content students seemed to engage more with (e.g., places where students paused frequently and/or accessed repeatedly) in detail for two selective videos. A special pattern of students’ accessing behaviors near the embedded interaction points observed from an exploration of students’ invideo accessing behaviors for all videos in the course will also be discussed.

Before we proceed, we would like to remind the readers that in this paper terms like “accessing,” “engagement,” etc., represent student interaction with the videos as recorded by the video player. Because of the limitation of data available we only have access to the time stamped clickstream data recorded by the video player but cannot monitor how attentive students were when the videos were playing; the *extent* to which students were engaged when playing a video is beyond the scope of this study. In order to gain more insight into students’ video accessing behaviors, student responses on an end-of-course survey will also be explored. In the remainder of the paper, we first discuss existing studies relevant to student engagement with videos, and then provide a detailed description of the course structure, student background, and the instructional videos. We then describe our research methodology, report findings from the study, and conclude with possible future work that may have the potential to help improve student learning in the course.

## II Background

With the increasing use of videos in educational settings, student learning via instructional videos (whether presented as supplemental material or as the primary means of delivering course content) has gained interest to the educational community. One thread of research commonly explored by educators is the extent to which students make use of these resources [12] and how video accesses correlate to student performance in the course [17,18]. This research has produced mixed results. For example, a study in which web-based multimedia learning modules (MLMs) were introduced in an introductory physics course at University of Illinois at Urbana Champaign [9] has shown that in the semester when MLMs were provided as part of the prelecture assignments, there was a significant improvement in student performance on prelecture conceptual measures compared to that from previous semesters in which MLMs were not provided. When students were separated into groups based on ability levels, it was found that students who viewed the MLMs consistently outperformed the nonviewers in all ability groups. A study in a European Law course [17] in which video recordings of in-class lectures were provided as supplemental materials showed that when controlled for important factors such as GPA and time spent studying, the number of postclass video accessing is positively correlated to students’ course grade. On the other hand, a study [18] in two calculus courses showed that the tendency to both attend in-class lectures and access online recording of previous lectures is negatively correlated to students’ final grade. Similar mixed results are not only reported at the video- accessing session level, but also at the detailed interaction level. For example, researchers from the University of Toronto at Scarborough have found that while the usage of pauses and seeks in videos is related to higher exam scores in an introductory psychology course, usage of pauses is negatively correlated to the final grade in a calculus course [18]. These studies suggest that many factors can influence the relationship between video accessing and student performance. For example, while repeated access allowed by videos may help improve students’ understanding of the materials, it is also possible that students who need to access the videos most are those who have greater difficulty in the course. Moreover, factors such as the types of content covered in the videos (e.g., communication of concepts vs procedural skills to solve problems), and the learning strategies used by students (e.g., focusing on the meaning of the tasks with the goal of maximizing understanding vs focusing on the concrete aspect of the tasks with the goal of avoiding failure with minimum time and effort) can all affect the relationship between video accessing and student performance [18]. A study that explores students’ understanding and perception of content presented in a lecture regarding vibrations, waves, and sound also points out that prior knowledge can have an effect on a person’s interpretation of the knowledge transferred in a lecture presentation [19].In particular, experts and students were asked in this study to indicate if the answers to a list of physics questions were addressed in the given video lecture. The experts not only indicated that the questions have been addressed in the video more frequently, but also believed that the questions were addressed more thoroughly than the students did. As this study shows that students may interpret the information differently than what the instructor in the video intended, it is possible that a deeper look into students’ video accessing behavior can also help shed light on how students learn from videos. To our knowledge, studies that reported on how video accessing relates to student learning in an introductory physics course [9,20] are still currently limited, especially when the videos were provided as major resources that introduce students to important concepts and skills in the course. Moreover, few studies in physics have examined how students access videos on the detailed interaction level. The current study attempts to explore these issues in the context of our on-campus, flipped course setting.

In addition to the connection between video accessing and learning outcomes, student engagement of lecture videos [12,13] itself is also of interest because understanding how students use lecture videos can help instructors attend to specific parts of the videos to assist students in learning. If peaks are observed in students’ accessing trajectory of in-video content, places where these peaks occur may warrant instructor follow-up as this activity may indicate student interest, confusion, or some other significant reaction to this point in the given video. Similarly, if students commonly disengage with specific points of a video, and the follow-up investigation shows that students do not have a full understanding of the concepts discussed in those sections, changes to the video and/or other relevant course activities may be made to help students benefit from it more. A study of user interaction with hundreds of videos from four edX MOOCs [13] identified 5 patterns for peaks in students’ in-video content accessing, which are most often due to visual transitions such as a video beginning new material, students returning to watch missed content, students pausing and leaving the video to complete a tutorial step, or students replaying a small segment of the video surrounded by visual transitions both before and after. The same study also found that students accessing videos often do not complete videos. A predicted dropout rate of 53% or more is obtained for videos exceeding 5 min long using a linear regression model [13]. Similar work on student engagement with videos has led to a series of suggestions on future video production [13,15]. For example, videos should be short, and should avoid abrupt transitions. Moreover, interactive links or timelines help students find common points of interests in their re-access of videos. We note that in the prior study discussed here, dropout is defined as the last point of access in a video accessing session, regardless of what students might have accessed or skipped through earlier in the given video. Since students can access the video nonlinearly, in our current work, the relation between video length and student-video interaction is explored from a slightly different angle in terms of what fraction of in-video content is accessed by the students. In addition, we explore this issue further by comparing the results between videos of different content types.

## III Course Structure, Student Background, and the Instructional Videos

### A Course structure

The calculus-based introductory mechanics course explored in this study was taught with the “Matter and Interactions” [21] curriculum in which students learn to solve mechanics problems starting from three fundamental principles: (i) the momentum principle (Newton’s second law), (ii) the energy principle, and (iii) the angular momentum principle. This curriculum, which places an introductory physics course in a modern context and emphasizes important scientific practices like modeling and computation, has been offered at the Georgia Institute of Technology in a large-lecture course for many years. In order to engage students in more group collaborative work during the in-class time, a “flipped” version of this course was offered starting in Fall 2013. In this flipped course, the traditional lectures were substituted by online videos. In particular, a number of lecture videos we created were assigned each week. Students were instructed to watch these online videos before coming to class for group problem-solving work. They were also instructed to perform laboratory activities individually at home by observing the motions of objects in their own surroundings, analyzing these motions through video analysis [22-24], and modeling the motions via Python programming language. A total of five laboratory activities were implemented in this course. The first four labs feature constant velocity motion, falling motion with drag, planetary motion, and spring motion in two dimensions, respectively. The 5th lab was a “choose your own adventure” lab, in which students were expected to take advantage of what they learned in the course to explore any kind of motion that was of interest to them. For each laboratory activity, students need to produce a short video report detailing their work and their results. Each laboratory activity had a 2-week cycle. In the first week, students perform the laboratory activity and create a lab report. In the 2nd week, students peer evaluate each other’s reports. Other out-of-class activities students participated in involved homework assignments, textbook readings, and online forum discussion.

In total, 161 students enrolled in this flipped version of the introductory mechanics course, and they were split into sections of approximately 25 students each for the inclass activities. Every week, students met in class with the instructor and the TAs in their own section for 3 h. About 2 h were spent on group problem solving in which students worked on tutorial-style problem-solving worksheets with 2 or 3 peers at their table. During this period, the instructor and TAs circulated within the class to interact with the students and to assist students with their work when needed. About 1 h of class time was spent on lab presentations. In this lab-presentation period, students practiced presenting their laboratory work to their peers in the form of either a draft report (during the first week of the lab cycle) or a final report (during the 2nd week of the lab cycle). The lab presentation section was led by a teaching assistant or the course instructor who would provide feedback and guide discussions among the presenter’s peers about how to best meet the goals of their presentations. After students participated in the in-class sections, which were held Monday to Thursday, a weekly quiz was held on Friday to allow students to check for their understanding of the materials. These quizzes were conducted in a proctored setting and administered on computers. There was one written midterm exam and one written final exam during this 17-week-long course.

### B Student background

All 161 students enrolled in this flipped course were STEM majors, for whom an introductory physics course is a requirement. Most of the students were between 18 and 24 years old, and slightly more than half of the students were female. Based on a voluntary background survey responded to by three-quarters of the students, only 8% of the survey participants had never taken any physics courses before in high school or in college. The Force and Motion Conceptual Evaluation [25] was implemented as an extra credit assignment at the beginning of the semester to evaluate students’ conceptual understanding when they first entered the course. One hundred and sixteen students responded to this assignment, and they had an average score of 36.4%. (We note that there was no statistically significant difference between students who responded to this pre-FMCE assignment and those who did not, based on their performance on the final exam.)

### C Instructional videos

As described earlier, students were required to participate in lectures and laboratory work outside of the classroom at their convenience. A total of 78 instructional videos were assigned throughout the whole semester to assist students with these at-home activities. These 78 videos can be grouped into two categories: 64 of them were “lecture-oriented videos” that introduced students to specific physics concepts and/or problem solving skills. These videos covered content that an instructor would typically discuss in lectures. Most of these lecture- oriented videos were whiteboard animated with the intent to attract and to hold the interest of students. The other 14 videos were “laboratory videos,” which were tied to skills and concepts necessary for successfully completing the at-home laboratory activities. In particular, 8 of them were “lab-specific videos” that provided specific information relevant for completing a particular lab. Six of them were “supplemental laboratory videos”, which introduce general concepts, skills, or techniques that were generally useful for all laboratory activities. Table I provides the titles of a few example videos in each group. A breakdown of the major physics topics discussed in this course and the corresponding number of lecture-oriented videos for each topic are shown in Table II. These 78 videos were typically 5–20 min long, with clicker questions that addressed important concepts embedded as interaction points in more than half of these videos. Students can access the videos by streaming them online as well as downloading them. These 78 videos make up the data explored in the rest of this paper.

**Table I:** Examples of videos in each category.

Category	Examples
“Lecture-oriented videos” (*N* = 64)	• Vectors in 1D
	• Newton’s second law
	•Spring potential energy
“Laboratory Videos” (N = 14)	*Lab-specific*: (8 videos, assigned between week 1 and week 8)
	• Video Analysis of Constant Velocity Motion: How to use Tracker
	•Creating a Computer Model of Constant Velocity Motion
	•Black Hole Lab Introduction
	*Supplemental:* (6 videos, assigned in the first two weeks)
	•Installing VPython
	•Using a Spreadsheet
	•Recording Observations on Video
	•Creating a Good Video Lab Report

**Table II:** Major topic breakdown for lecture-oriented videos and the corresponding number of lecture-oriented videos for each topic.

Topic	Number of lecture-oriented videos
1. Overview of the overarching physics ideas in mechanics and useful mathematical concepts	7
2. Using forces and Newton’s second law (momentum principle) to predict future motion	17
3. Finding forces from motion observations	6
4. Energy principle and relevant energy concepts	15
5. Multiparticle systems	11
6. Angular momentum principle	8

## IV Methodology

To provide a sense of how analysis related to student-video interaction is conducted, we first present a single video-streaming session by a single student. Then, analysis of student aggregate behaviors, which is the focus of this paper, will be discussed. Details about the end-of-course survey, which provides triangulation for understanding students’ video accessing behaviors, will be presented at the end of this section.

### A Example from a single streaming session by a single student

**Fig. 1:**
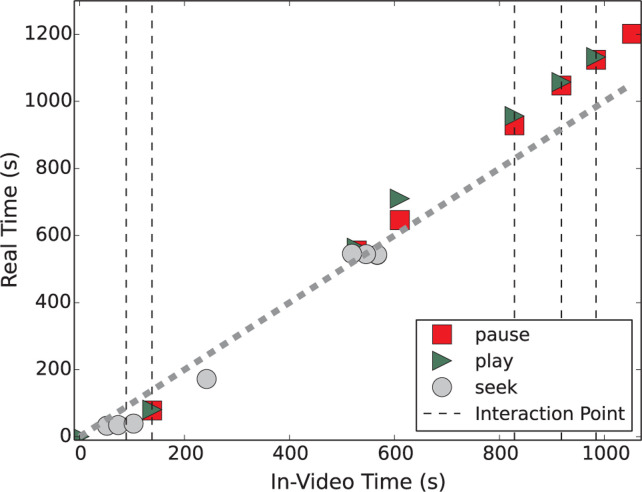
The “accessing trajectory” of a single student. The dashed diagonal line represents where video time and real time are identical.

**Fig. 2:**
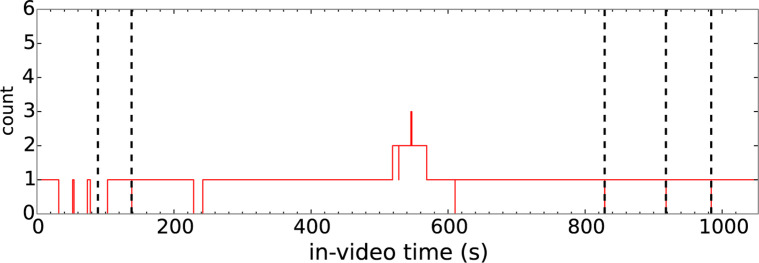
Time series indicating the number of times student 3 accessed a given second in video 15 during that student’s 5th accessing session (*A_ijkt_* with *i* = 3, *j* = 15, *k* = 5). In this session, student 3 accesses most of the video once while accessing 519-567 s multiple times.

Figure 1 shows an example of a student’s video streaming behavior. In particular, this was the fifth time student No. 3 accessed video No. 15 through online streaming; this was also the first time this student accessed the given video to its conclusion. Three types of events recorded of student interaction are shown in this figure: plays, pauses, and seeks. Plays (represented by green triangles in Fig. 1) and pauses (represented by red squares) can be manually generated by the student or autogenerated by the video player. For example, there will always be a play at the beginning of each video and a pause at the end. Seeks are recorded when a student clicks on the scrubber below the video to skip to a different point in the video. At the time when our course was offered, Coursera only recorded when or where a “seek” event ended, but not when or where it started. Therefore, only seek ends are presented in Fig. 2. However, an estimation of the portion of video accessed before the seek event happened can still be achieved using the playback rate and the time elapsed between the previous event and the seek event of interest. A detailed discussion of how such estimation is performed can be found in the Appendix. When the video player reaches an interaction point (represented by the vertical dashed lines in Fig. 1), a pause is autogenerated, and a new window pops up asking students to complete a task (e.g., downloading a supplemental file or answering a question.) When students exit the interaction point to access the rest of the video, a play will be recorded. On Coursera, several playback rate options (from 0.5× to 2×, in increments of 0.25) are available so that students can watch the video at their preferred rate. In this paper, we refer to the timeline in the video (like the timeline in a YouTube player) as “in-video time.” The dashed diagonal line in Fig. 1 represents where “in-video time” equals “real time.” Thus, if the slope between a play event and the subsequent pause event is larger (smaller) than the slope of the dashed diagonal line, the playback rate students used is smaller (larger) than normal playback of 1.0×. This video is 1052 s long (~17.5 min).

For each student-video interaction, plots like Fig. 1 provide an “accessing trajectory”, that is, a snapshot of how each student interacted with the video. Beginning from the origin of Fig. 1, this student played the video for approximately 32 s in real-time and then began to seek through the video. Because the video did not pause at the first interaction point (represented by the first vertical dashed line), this provides evidence that the student skipped past this interaction point. The video autopaused at the second interaction point, and playback was resumed ~2.7 s later. The student then played the video for ~91 s, skipped slightly ahead to time 242 s in the video, and then continued playing ~370 s before skipping to pause at time 528 s in the video. The student then played the video for another ~82 s until pausing for ~64 s at time 610 s in the video. Subsequent pause-play pairs correspond to the remaining three interaction points in the video. The majority of play-pause pairs in Fig. 2 have a slope equal to that of the dashed diagonal line, suggesting that this student watched the video with normal playback rate (1.0×) most of the time.

### B Analysis of students’ aggregate video-accessing behaviors

For each video-streaming session, an accessing trajectory similar to that discussed above can be retrieved from the data. With these accessing trajectories available, we can explore how students as a group accessed videos. To facilitate discussion of how our analysis is performed, we first introduce a matrix *A_ijkt_*, with the indices corresponding to the following:

- *i*, student ID (it takes values from 1 to *N_s_*, where *N_s_* represents the total number of students. In our study, *N_s_* = 161),
- *j*, video number in the assigned order (it takes values from 1 to *N_v_*, where *N_v_* represents the total number of instructional videos in the course. *N_v_* = 78),
- *k*, index of streaming sessions, i.e., the kth time a given student *i* clicked on the link for video *j* to access its content through online streaming (*k* takes values from 1 to *N_a,max_*, where *N_a,max_* represents the maximum number of times a student in our course has clicked on the link for the same video to stream it repeatedly. In our study, *N_a,max_* = 25), and
- *t*, in-video time in seconds (*t* takes values from 0 to *t_j_*, where *t_j_* represents the length of the given video *j* in seconds. While Coursera records data in the millisecond accuracy, for the purpose of this paper, we use 1 s as the sampling rate interval for simplicity);

For each video-streaming session [i.e., a given set of (*i, j, k*)], we construct a matrix representing how many times student *i* has accessed the content at in-video time *t* of video *j* during the *k*th time she streamed this video online; a value of 1 is added to element *A_ijkt_* every time the designated point of the video was accessed; otherwise, a value of 0 is assigned.

Figure 2 presents a slice of the matrix *A_ijkt_* for the example shown in Fig. 1 (with *i* = 3, *j* = 15, *k* = 5 since this was the 5th time student 3 streamed video 15). A detailed discussion of how the matrix *A_ijkt_* is calculated from the play, pause, and seek data recorded can be found in the Appendix. If a given student *i* has never accessed a given video *j* through online video streaming, ∑*_kt_A_ijkt_*=0 On the other hand, ∑*_kt_A_ijkt_*≠0 means that the student has clicked on the link for video *j* at least once to access its content online. The total number of online video-streaming sessions of video *j* initiated by student *i* (TS_*ij* from streaming_) can therefore be easily obtained from the matrix *A_ijkt_* with the following definition:

$${\mathrm{TS}}_{ij\;\mathrm{from}\;\mathrm{streaming}}=\left\{\begin{array}{ll} \max\left(k\right) & |\sum_{t}A_{ijkt}>0\\ 0 & \mathrm{if}\;\;\sum_{t}A_{ijkt}=0\;\mathrm{for}\;\mathrm{any}\;\mathrm{given}\;k \end{array}.\right.$$

We note that in addition to streaming videos online, students could also download videos to access them offline. A total of 17 092 cases of video streaming and 145 cases of video downloading were recorded in this course. Because of the types of data available, the matrix *A_ijkt_* is constructed only for the former cases of online video streaming. For the latter cases of video downloading, another matrix *D_ij_* was constructed to represent how many times student *i* has downloaded video *j*. *D_ij_* can be greater than 1 because some students were found to download the same video multiple times.

Using *A_ijkt_*, TS_*ij*_ from streaming, and *D_ij_*, we can define a few more quantities to represent students’ video accessing behaviors from different aspects. In the following, we present the meanings of these quantities. The mathematical definition for how to calculate each of these quantities from matrices *A_ijkt_*, TS_*ij* from streaming_, and *D_ij_* can be found in the Appendix.

(1) TS_*ij*_ (Total number of sessions): how many times student *i* has clicked on the link for video *j* to either access its content online or to download it. In other words, TS_*ij*_ is the sum of TS_ij_from streaming and *D_ij_*,
(2) *C*(*V*) (Complementary cumulative distribution function of the amount of unique videos accessed by students): What fraction of students have accessed more than a certain proportion of unique videos in the course. Here *V* represents the fraction of unique videos accessed, which ranges between 0 and 1. *C*(*V*) at *V* = 0.5, for example, indicates the fraction of students who have accessed more than 50% of the videos (i.e., 39 unique videos out of 78) in the course. When further separating the videos based on different content type, similar fractions *C*(*V*)_lec_ and *C*(*V*)_lab_ can be defined for lecture-oriented videos (with total *N* = 64) and laboratory videos (with total *N* = 14), respectively.
(3) FS_*j*_ (Fraction of students accessing a given video): out of all 161 students in the course, what proportion of students have ever accessed the given video *j* through either online streaming or downloading. FS_*j*_ ranges between 0 and 1.
(4) TA_*ijt*_ (Total number of accesses for specific in-video content by a single student): for a selected video *j*, how many times has student *i* accessed the content at *t* second of the given video. Because of the availability of data, this variable and the next variable are constructed only for cases of online video streaming. The few cases of video downloading [26] were excluded in such a type of analysis.
(5) FSE_*j*_ (Fraction of students accessing “almost” the entirety of video *j*): out of all the students who accessed a selected video *j* through online streaming, what proportion of them has accessed “at least 99%” of the content in the given video. Here, by “accessing” at least 99% of the content, we mean that at least 99% of the given video has been “played at least once” when combining all the accessing sessions made by the given student of the given video. In our analysis, the criterion of accessing the video “almost” entirely instead of 100% completely is applied. This is because in some situations students may think they have received all the information presented in a video even though strictly speaking not every single second of the given video has been accessed. For example, students may skip the first few seconds of each video because they find the opening music irrelevant to physics. In addition, students may require less time than provided to digest the information presented. Therefore, they may stop a video one or two seconds earlier than its official ending when only static footage but no audio was presented at the very end. In this paper, we set the criteria of accessing almost the entirety of a video as accessing at least 99% of the given video. To ensure that the results in this study are not significantly influenced by the exact criteria used, we have also performed the analysis with several different criteria for accessing almost the entirety of a video (e.g., skipping less than 1 s of the video, skipping less than 5 s of the video, accessing at least 80% of the given video, etc.) All the findings reported in this paper remain qualitatively the same regardless of the criteria used.

We note that the first three quantities presented here [i.e., TS_*ij*_, *C*(*V*), and FS_*j*_] focus on whether students have ever clicked on the link for a given video to download it or to access its content online. They do not take into account the amount of in-video content accessed by students within each video-accessing session. On the other hand, the last two quantities (i.e., TA*_ijt_* and FSE_*j*_) focus on the exact invideo content that was accessed by students. A short summary of the quantities discussed in this section can be found in Table III.

**Table III:** Short summary of the quantities used for describing students’ video-accessing behaviors. In this table, quantities without an asterisk are obtained by examining whether or how many times students have started a video-accessing session (i.e., clicking on the link for a video) regardless of the amount of in-video content accessed within each session. On the other hand, quantities denoted with an asterisk are obtained by focusing on the exact in-video content that was accessed by students. In addition, the ↓ symbol indicates quantities that are constructed for cases of video accessing through online streaming, while the ► symbol indicates quantities that are constructed for cases of video accessing through downloading.

Type	Quantity	Source of data	Meaning
Fixed quantities	*N_s_*		Total number of students in our study. *N_s_* = 161
	*N_v_*		Total number of instructional videos in the course. *N_v_* = 78
	*N_a,max_*	► only	Maximum number of times a student in our course has clicked on the link for the same video to stream it online repeatedly. *N_a,max_* = 25
Basic quantities	*i*		Student ID. It takes values from 1 to *N_s_*.
	*j*		Video number in the assigned order. It takes values from 1 to *N_v_*.
	*k*	► only	Index of streaming sessions. It takes values from 1 to *N_a,max_*.
	*t*	► only	In-video time in seconds
	*A_ijkt_*	► only	How many times student *i* has accessed the content at in-video time *t* of video *j* during her kth streaming session of the given video
	*D_ij_*	↓ only	How many times student *i* has downloaded video *j*.
Derived quantities	TS_*ij*_	► & ↓ combined	Total number of sessions: how many times student *i* has clicked on the link for video *j* to either stream it online or to download it.
	*C(V)*	► & ↓ combined	Complementary cumulative distribution function of the amount of unique videos accessed by students
	FS_i_	► & ↓ combined	Fraction of students that have ever accessed a given video *j* through either online streaming or downloading (out of all students in the course)
	TA_ijt_^*^	► only	Total number of accesses for specific in-video content by a single student: for a selected video *j*, how many times has student *i* accessed the content at *t* second of the given video (summed over all video-streaming sessions made of the given video by the given student)
	FSE_j_^*^	► only	Fraction of students accessing almost the entirety of video *j* (out of all the students who has ever accessed the given video through online streaming)

### C End-of-course survey

At the end of the semester, students were asked to fill out a voluntary online survey that consists of 14 sets of questions to help instructors better understand student experiences in the course. Students were asked in this survey, for example, to rate their experience with each course component (including the online instructional videos, the laboratory activities, homework, on-campus sections, etc.). Students were also asked in this survey to report the time they spent each week on each component of the course, to identify course features that were valuable in helping them learn physics, and to report their feelings about this new course format. Most of the survey questions are presented in Likert-scale format, but there are also a few free-response questions encouraging students to share any comments, suggestions, or stories they had for either the overall course or specific course component(s). Three- quarters (121 out of 161) of the students responded to this end-of-course survey. There was no statistically significant difference found on students’ final exam performance between those who responded to the survey and those who did not. (Average score on final exam was 68.1 for the former and 68.9 for the latter).

## V Results

### A The extent to which students access videos

**Fig. 3:**
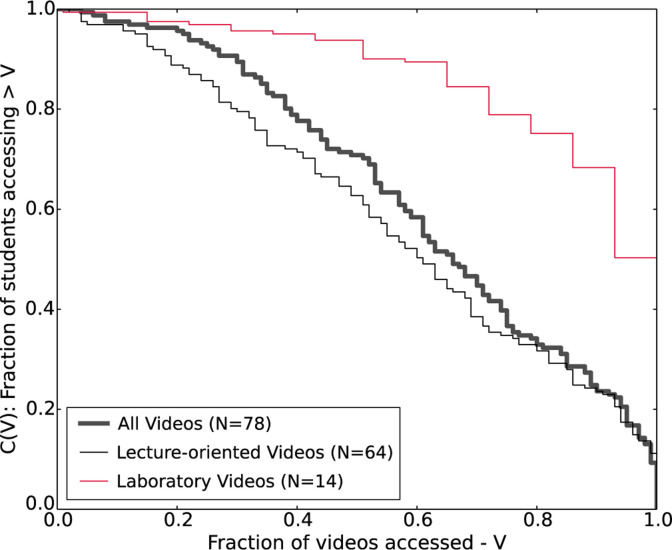
Complementary cumulative distribution function C(V) showing the fraction of students accessing more than a certain proportion of videos in each group. Students are much more likely to access most or all of the laboratory videos in comparison to the lecture-oriented videos.

The first major goal of this paper addresses the issue of to what extent students made use of the instructional videos online. Figure 3 presents the complementary cumulative distribution functions of the unique videos accessed by students for each type of videos [i.e., *C(V*), *C(V*)_lec_, *C(V)*_lab_ defined earlier in the methodology section]. Overall, 9% of students accessed all 78 videos, and half of the students skipped more than 35% of the videos. These results suggest that many students did not think it is necessary to view all of the videos exhaustively. A comparison between the fraction of students accessing lecture-oriented videos and the fraction of students accessing laboratory videos shows that laboratory videos were accessed more than the lecture- oriented videos. While more than half of the students accessed all the laboratory videos, only 11% of the students accessed all the lecture-oriented videos.

**Fig. 4:**
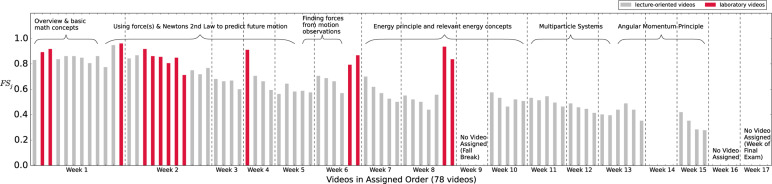
Fraction of accessing students (FS_*j*_) for each video. The lecture-oriented videos are represented in gray. The laboratory videos are represented in red. No videos were assigned in week 9, week 14, week 16, and week 17. Laboratory videos specific for the 1st, 2nd, 3rd, and 4th lab were assigned in weeks 1 and 2, week 4, week 6, and week 8, respectively. The 5th lab was a “choose your own adventure” lab, in which students were expected to take advantage of what they learned in the course to explore any kind of motion that was of interest to them. Therefore, no laboratory videos specific for this lab were provided. Weeks 1 and 2 also included supplemental lab videos concerning specific tools used in all lab activities instead of specific lab activities. Major topic breakdown for the lecture- oriented videos is indicated on the figure. The figure shows that students reduced their accessing of lecture oriented videos as time progressed while maintaining their access for laboratory videos.

Given that students did not access all videos, it is natural to ask whether a video’s placement in the course had an effect on its corresponding FS_*j*_ (i.e., fraction of students who has ever accessed the given video *j*). For each video, the time when it was assigned, and the corresponding FS_*j*_ are shown in Fig. 4. A majority of week 1 videos were accessed by more than 80% of the students. These videos provide an overview of the overarching mechanics ideas, present useful mathematical concepts, introduce students to the computational modeling tools used throughout the course, and discuss the first fundamental physics principle in the course (Newton’s second law). As the semester progressed, however, many students stopped accessing the lecture-oriented videos. For example, the fraction of accessing students dropped to an average of 54% for lecture-oriented videos related to the energy principle and relevant energy concepts, which were assigned in the middle of the semester. The fraction of accessing students dropped further to lower than 40% for the last few lecture-oriented videos about angular momentum principle assigned at the end of the semester. On the other hand, the fraction of students accessing laboratory videos did not seem to be affected by the videos’ placement in the course. Even for the last 2 laboratory videos, the fraction of students accessing them remained higher than 84%.

**Fig. 5:**
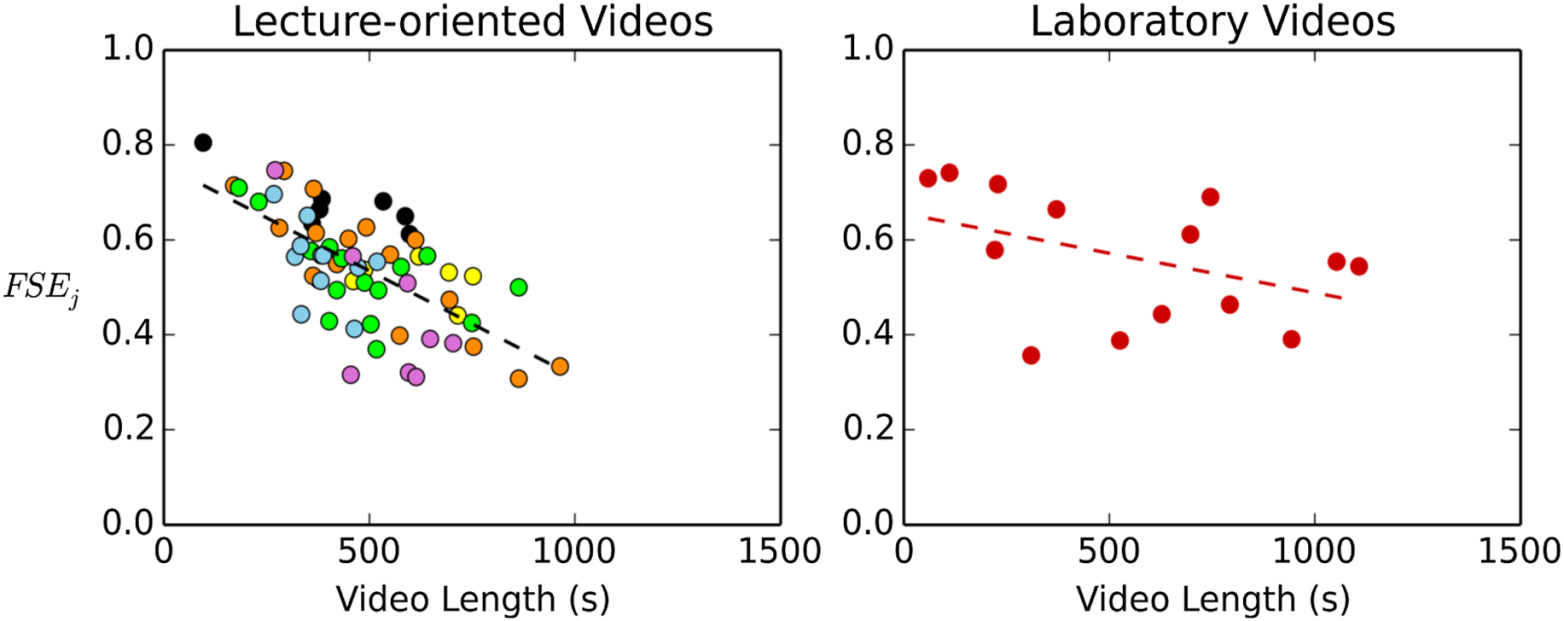
Video length vs fraction of students accessing more than 99% of the given video (FSEj). Each data point represents one single video in the course. The lecture-oriented videos are color coded based on different topics, with black, orange, yellow, green, blue, and purple representing the 1st to 6th topics presented in Table II, respectively. The trend lines for all lecture-oriented videos combined and all laboratory videos combined are *y =* —0.000 447*x* + 0.758 and *y =* -0.000 167*x* + 0.655, respectively. The figure shows that the fraction of students accessing the entirety of a video decreases when videos become longer in length. Moreover, this trend is more prominent for the lecture-oriented videos than the laboratory videos.

With an understanding of whether the students clicked on the links to access videos, we now take a step further to investigate how students interacted with the videos after they clicked on the video link. In particular, we focused on whether students would access the video completely once they decided to access the given video. Figure 5 shows the fraction of students who accessed at least 99% of a given video (FSE_j_), and how this quantity relates to the length of the given video. When averaged over all videos in the group, the mean FSE is 0.54 for lecture-oriented videos and 0.56 for laboratory videos. This suggests that when students accessed videos, only about half of the time they would access almost the entirety of the video. Moreover, when lecture-oriented videos become longer in length, the fraction of students accessing almost the entire video decreases. We observe this trend starting at the beginning of the semester (when videos are dominated by overarching physics ideas and useful mathematical concepts) and continuing over the rest of the semester (when videos contain more varied physics content). In addition, a comparison between the lecture-oriented videos and the laboratory videos shows that the fraction of students accessing almost the entire video decreases with a slope of -0.00045 /s for lecture-oriented videos. For the laboratory videos, the fraction of complete-accessing students did not decrease as much with video length (slope = —0.000 17/s). A higher interest in the laboratory videos than lecture-oriented video was, therefore, not only observed in the video-accessing session level but also at the detailed-interaction level.

Overall, all the findings above show that students in our course engaged in laboratory videos more than the lecture- oriented videos. We note that in this flipped course, although students were instructed to watch the assigned videos before coming to the in-class section, they were not graded on whether or not they accessed the videos. Without direct grade incentives, students’ video accessing behaviors were most likely driven by their personal interests (e.g., their judgment of the value of a given video). In this course, no instruction on how to perform the laboratories was provided in class. Therefore, students may have high interests in the laboratory videos because these videos are considered the major resource helpful for completing the laboratory assignments. On the other hand, the lecture- oriented videos may not be viewed as the only resource helpful for learning about mechanics. The end-of-course survey results in Table IV show that while many students believed the instructional videos were informative, easy to understand, and stylistically engaging, the students broadly considered in-class problem-solving sessions and homework to be more valuable than the online lecture videos. When asked to describe the factors that prevented them from participating in the online instructional videos, one student noted *“I am able to learn the material by remembering high school physics and doing the practice problems during lecture/lab [i.e., the scheduled in-class period], so the videos are unnecessary unless I really don’t have a grasp on something”* Another student noted *“Lecture videos were less helpful than just doing work packets in lab”* If students felt that other course components were more beneficial for learning the materials, and (or) that their prior exposure to physics content was sufficient to learn the materials, students may not be interested in accessing the videos. In addition, the fact that the quizzes in the course were conducted on computers in which 100 submissions were allowed may have also contributed to the low number of accessing sessions for lecture-oriented videos. As a student responded in the end-of-course survey, *“I will be honest. I only watched the lectures during the beginning of the course. They were helpful, but there was little motivation to watch them especially with how easy the weekly quizzes were*.” If no direct grade incentives were provided, and if students do not see a direct relation between how accessing lecture-oriented videos can help improve their performance in class, they may be likely to gradually disengage in these videos.

**Table IV:** Percentage of students who agree or disagree with statements in the end-of-course survey regarding various course components. A few students did not answer all the questions on the survey so the percentages do not always add up to 100%.

Statement	Disagree	Neutral	Agree
The videos were informative and easy to understand	9.2	16.0	70.6
The videos’ style and appearance was engaging	3.4	18.6	73.7
The videos were valuable in helping me learn physics	24.0	21.5	52.1
The homework assignments were valuable in helping me learn physics	4.2	7.5	88.3
The on-campus problem solving sections were valuable in helping me learn physics	3.4	5.9	90.8

### B Relation between video accessing and student performance

With a basic understanding of students’ video accessing behavior, we now shift focus to the correlation between video accessing and student performance shown in Table . Here several different measures are used to describe students’ video accessing behavior, such as the number of videos accessed, the total time spent accessing videos, total length of videos accessed by students, and the frequency of students’ interactions with the video player. Similarly, different measures are used to describe students’ course performance, such as final exam score, lab grade, and post FMCE score. No strong correlation was found between any video accessing measure and any course performance measure. This result is similar to our experiences from several semesters of traditional lecture-style introductory mechanics courses held at the Georgia Institute of Technology, in which weak correlation (*r =* 0.33) between student attendance of lecture and student performance on the final exam was found. Table V also shows that there is no strong correlation between students’ video accessing behaviors and their incoming GPA or pre FMCE score, either.

**Table V:** Relation between video accessing versus student performance or incoming background. The Pearson Correlation Coefficient (*r*) for each case is provided. Overall, students’ video accesses do not show strong correlation to their course performance or incoming background.

Video accessing versus student performance or incoming background	*r*
Number of videos accessed vs score on final exam	0.16
Total time spent accessing videos vs score on final exam	0.00
Total unique in-video time accessed vs score on final exam	0.23
Frequency of pause interactions (i.e., total number of pauses divided by total unique in-video times that have been accessed) vs score on final exam	0.12
Average fraction of in-video content accessed (i.e., total unique in-video time accessed divided by the sum of video lengths of all videos accessed) vs score on final exam	0.25
Percentage of videos accessed almost completely (i.e., ≥99% in-video content accessed) vs score on final exam	0.19
Time spent accessing laboratory videos vs grades received on related lab	0.11
Number of videos accessed vs post FMCE	0.01
Total time spent accessing videos vs post FMCE	0.14
Total unique in-video time accessed vs post FMCE	0.03
Number of videos accessed vs incoming GPA	0.28
Total time spent accessing videos vs incoming GPA	0.16
Number of videos accessed vs pre FMCE	0.03
Total time spent accessing videos vs pre FMCE	0.00
Total unique in-video time accessed vs pre FMCE	0.07

### C Detailed student interaction with videos

**Fig. 6:**
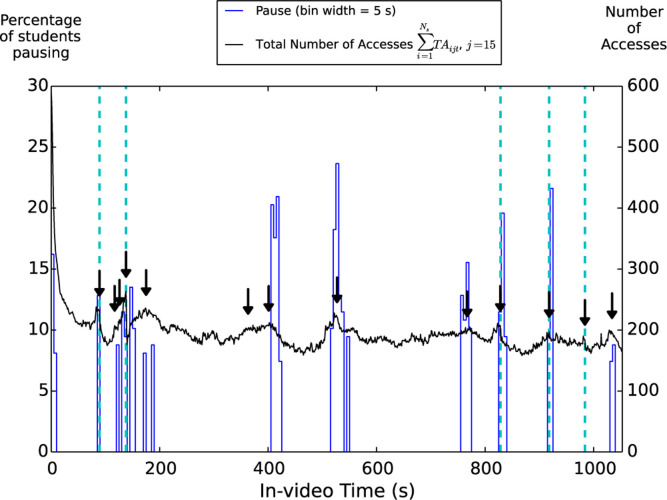
Clickstream analysis of video 15. This video, entitled “Creating a Computer Model of Constant Velocity Motion,” was accessed by 148 unique students in total. The black line represents the number of times each particular point of a given video was accessed by students (i.e., $\sum_{i=1}^{N_{s}}{\mathrm{TA}}_{ijt}$, where *j* = 15.) The blue line represents the percentage of unique students who has ever paused within each 5 s time window. If multiple pauses were made by the same student within a 5 s window, this student was counted only once in the given window. Since we are interested in student-generated pauses, the computer-generated pauses both at the interaction points and the very end of the video have been taken out. In order to help identify peaks, pauses are not plotted for all the 5 s time windows. Instead, only cases for which the percentage of students pausing is greater than or equal to 2 median absolute deviations [27] above the median value of all cases (i.e., 2σ + median), which in this case corresponds to 7.4%, are shown in the figure. The interaction points in the video are indicated by the vertical dashed line. Places where “actions” are demonstrated in the video are indicated by the black arrows. The figure shows that student engagement increased during these action points. A description of the action each arrow represented is included in Table VI.

As presented in the previous section, in our course, students’ performance did not correlate with their video accesses. A prior study [18] suggests that if students had a surface approach to learning that focused on the concrete aspects of a task rather than the meaning of the task, then more engagement with instructional videos would not necessarily lead to better performance. In order to get a deeper insight into how students in our course interacted with the videos in detail, an exploration involving the most- accessed laboratory video (*j* = 15) and the most-accessed lecture-oriented video (*j* = 11) was conducted. In particular, we manually inspect the content in these videos to identify the type of content (if any) students in our course seemed to engage more with. The findings suggest that students in our flipped course seemed to engage more with content that provides *concrete* information useful for assignment completions. However, students may not engage as much with other content that is also considered important from an instructor’s point of view. For example, Fig. 6 shows the total number of accesses made by students for each particular second in the most-accessed laboratory video (i.e., $\sum_{i=1}^{N_{s}}{\mathrm{TA}}_{ijt}$, *j* = 15.) In addition to the total number of accesses $\sum_{i=1}^{N_{s}}{\mathrm{TA}}_{ijt}$, the number of unique students who accessed a particular point of the video more than once has also been plotted for comparison in our data analysis process. This is done to identify the peaks in $\sum_{i=1}^{N_{s}}{\mathrm{TA}}_{ijt}$ that were actually generated by intentional student access and not, for example, by a malfunctioning video player. Since “the total number of accesses” and “the total number of unique students with repeated accesses” display similar trends, only the former is presented here. In addition to the number of accesses, we took pausing as another indicator of student engagement, since we would expect a student to pause a video to take notes of important information or to repeat important passages in the video. Places where students paused the most are plotted in Fig. 6. Overall, the high-frequency noise shown in the number of accesses in Fig. 6 suggests that students skipped frequently through this video. Moreover, Fig. 6 indicates that in this laboratory video, which teaches students how to construct a computational model for constant velocity motion from a starter Python code provided, high student access occurs at places where *actions* useful for completing the corresponding laboratory assignment are demonstrated on the screen. (See Table VI for a description of the *action* each arrow represented in Fig. 6.) Here we define action as a situation in which a physical act (such as downloading a starter python code, entering a parameter value required, running the code to check for the result) from the student is required or explicitly recommended in the video for the completion of the assignment. If, for example, the video discusses an important parameter in the Python code, but there is no need to change the default value in the starter code for that parameter, it does not constitute an action defined here. Figure 6 shows that students not only accessed these “action” sections more frequently but also paused a lot in these sections. On the other hand, sections in which the instructor discusses the physics concepts behind but no modification of the code is required are less engaged by the students. For example, from 600 to 673 s, the instructor points out that in the iteration loop where each time the motion would be predicted a small time ∆*t* into the future using Newton’s second law $\left({\vec{F}}_{\mathrm{net}}/m\;=\;∆\vec{v}\;/∆t\right),\;\;∆t$ needs to be chosen such that it is much less than the typical time scale of the motion being observed. In the example used in this video, the ball is shown moving for about 1 s. The instructor discusses how the starter code’s default time step (∆*t* = 0.01 s) is suitable for describing the ball’s motion during this interval. He further points out that it is not necessary to set At to be equal to the time between frames of the recorded motion (which many students tended to do in our experience), especially when the time between frames is large. While this discussion contains important physics behind the computational model, students did not seem to engage with this section as much as they do with the action sections.

**Table VI:** 

In-video time	Actions demonstrated or suggested in in the video
89 s	Watch a pep talk on coding and computing using the link provided
117 s	Launch VIDLE (a software application that provides the setting where codes will be written and run)
126 s	Open the starter code
138 s	download the starter code using the link provided if students had not done so
175 s	Run the unedited starter code to make sure it runs without error
363 s	Rotate the orientation of the visualization window by right clicking and dragging
401 s	Edit a line of code so that it correctly represents the mass of the object 527 s
Edit the codes so that the initial conditions of the object are correctly included	767 s
Edit a line of code to specify the iteration limit of the while loop
828.5 s	Edit a line of code so that it correctly represents how velocity update should be performed
918 s	Edit a line of code so that it correctly represents how position update should be performed
984 s	Edit a line of code to put in the net force for the motion under study
1034 s	Run the program

**Fig. 7:**
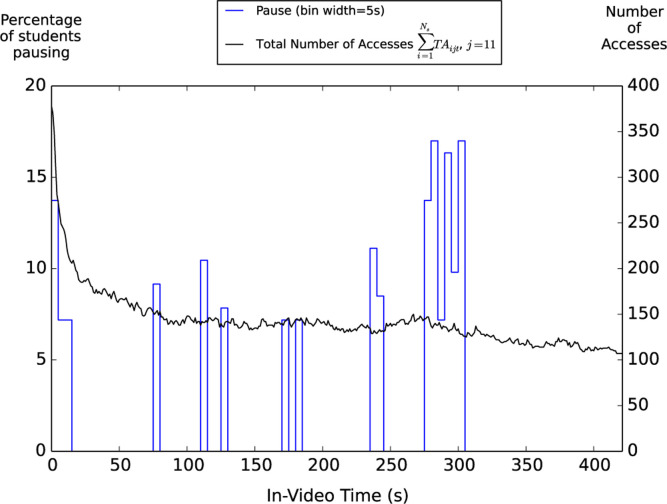
Clickstream analysis of video 11. This video, entitled “Newton’s second law”, was accessed by 153 unique students. The black line represents the number of times each particular point of a given video was accessed by students (i.e., the value at in-video time *t* was obtained by $\sum_{i=1}^{N_{s}}{\mathrm{TA}}_{ijt}$, where *j* *=* 11). The blue line represents the percentage of unique students that have ever paused within each 5 s time window. To help identify common pause peaks, only cases for which the percentage of students pausing is greater than or equal to 2 median absolute deviations above the median value of all 5 s windows *(la +* median, which in this case corresponds to 7.2%) were shown in the figure. Since we are interested in student-generated pauses, automatic pauses that occurred at the very end of the video have been taken out.

Figure 7 shows the total number of accesses and the percentage of students pausing at various in-video time for the most-accessed lecture-oriented video (*j* = 11). For this video entitled “Newton’s second law”, while there is no strong peak in the number of accesses, pause peaks are generally found in regions that discuss *concrete* information or techniques useful for problem solving (such as 7580 s where the instructor discusses the dimension and SI unit of mass; 110-115 s where the instructor discusses the fact that forces have magnitude (i.e., how strong?) and direction (i.e., which way?); 235-245 s where the instructor discusses a tip for finding net force; 275-305 s where Newton’s second law and the relevant units are presented; see Table VII for a full description of the video content that corresponds to each major pause peak in Fig. 7). While these sections contain important information, other sections also convey important concepts associated with Newton’s second law. For example, from 308-324 s, the instructor discusses the important role of Newton’s second law $∆\vec{v}\;/∆t\;=\;{\vec{F}}_{net}/m$ by pointing out *“Newton’s second law is an amazing statement which relates something we can obtain just by directly watching our object moves during some time interval, to, very different quantities that we cannot generally get at just by looking at our object.*” From 363–417 s, the instructor discusses the epistemology behind Newton’s second law and explains what makes Newton’s second law a law by saying *“First, Newton’s second law is a law because it tells us a secret of the universe. It’s not obvious that quantities that describe motion should be related to object properties with the influence of the surroundings this particular way. Second, Newton’s second law is a law rather than somebody’s opinion or a wild guess, because it is a statement about nature that has withstood the tests ofcountless experiments with moving objects over a wide variety ofconditions. This means you don’t have to believe it just because I told you so. You’ll have plenty of opportunities to check this for yourself”* However, students accessed and paused less at these sections, suggesting that students may not engage as much in such type of information that is typically less explicitly manifested in a problem solving process despite the importance of this information in the construction of a solid understanding of physics. In fact, when students were asked about their feedback for the instructional videos in the end-of-course survey, a common opinion expressed by the students is that the videos would be more helpful if they focus on the applications more. For example, a student points out that “*It would be helpful if the videos made it clearer which formulas were important. For example, at* *the end of each video all the different formulas introduced in the lecture could be written on the screen.*” Another student points out that “*a lot of the time they [the videos] explained the concepts really well. Which is great and all but they didn’t always address how to apply the concepts, which is all that really matters in this course. If you understand the concept, but you can’t do the problem, you still get the problem wrong.”* Responses mentioning “*more examples*” or “*more clicker questions*” are also frequently found.

**Table VII:** Description of the video content that corresponds to the major pause peaks in Fig. 7.

In-video time (s)	Description of video content
75-80	Dimension and SI unit of mass
110-115	Forces have magnitude (how strong?) and direction (which way?)
125-130	(When drawing an arrow to represent a force) the length of the arrow represents not a length but the strength of the force.
170-185	Finishing the statement that net force is the sum of all forces acting on the system and demonstrating how to perform vector sums to find the net force when each push or pull is represented by a vector.
235-245	3rd tip for finding net force: Objects do not exert forces on themselves (while info for the1st and 2nd tips is still on the screen; tip 1: count all forces from surroundings on system; tip 2: never count forces on the surroundings)
275-305	• Mathematical expression for Newton’s second law;
	• Discussion that both *F⇀*_net_ and Δ*v⇀* are vectors, i.e., they have direction and magnitude
	• units on both sides of the Newton’s second law equation
	• using dimensional analysis to find the unit of net force; a table summarizing the dimension and SI unit of force

In Fig. 7 and Table VII, it is also worth noting that the peak corresponding to mathematical manipulation (in this case dimensional analysis) has the highest number of students pausing among all the pauses. While instructors might have considered dimensional analysis to be no more important than other information (such as tips for finding net force), the former nevertheless shows more pauses (285-300 s) than the latter (230-245 s). From 230-245 s, several tips for finding net force are presented in the video (e.g., never count forces on the surroundings; do not include forces that the objects exert on themselves.) Although these tips were designed based on common student difficulties, students may not necessarily be aware of the importance of this discussion and may not feel the need to pause there as much as they do for places in which mathematical equations appear on the screen.

**Table VIII:** Example made-up data explaining how events would be recorded in clickstream (the first 3 columns [29]) and how matrix *A_jkt_* would be constructed (the last 3 columns) for the following scenario: a student watched the first 40 s of a video, clicked on pause, and then clicked on the scrubber below the video to jump to the point that corresponds to 100 s in in-video time. Then, she clicked on play to continue watching the video for another 20 s. In this example, the playback rate student used was 1.

Event	Real time (s)	In-video time (s)	Implication for elements in matrix A_ijk_
Play	0	0	A 1 is added to every element between *A*_*ijk*0_ and *A*_*ijk*40_
Pause	40	40	
Seek	41	100	None
Play	42	100	A 1 is added to every element between *A*_*ijk*100_ and *A*_*ijk*120_
Pause	62	120	

In addition to the findings from these two videos discussed above, an exploration of students’ in-video content accessing behavior of all 78 videos altogether indicates an interesting finding: the total number of accesses (i.e., $\sum_{i=1}^{N_{s}}{\mathrm{TA}}_{ijt}$) usually increased toward an interaction point and dropped immediately after the interaction point (see the 1st, 2nd, 3rd, and 5th interaction points in Fig. 6 for examples). In the 86 interaction points given in the videos throughout the course, 66 of them had a higher $\sum_{i=1}^{N_{s}}{\mathrm{TA}}_{ijt}$ compared to that of 5 s before, and 62 of them had $\sum_{i=1}^{N_{s}}{\mathrm{TA}}_{ijt}$ that was lowered by at least 10% at 1 s later. Moreover, a deeper look into students’ accessing trajectory shows that one reason why $\sum_{i=1}^{N_{s}}{\mathrm{TA}}_{ijt}$ drops immediately after the interaction point is that some students did not access the content right after the interaction point at all. When comparing the number of unique students accessing the information right before a given interaction point to the number of unique students accessing the content immediately after the same interaction point, it was found that the latter is generally smaller than the former. Moreover, a more than 10% drop in the number of unique accessing students was found in 20 of the interaction points, with the greatest drops occurring at interaction points placed close to the end of the video. In our videos, a question for students to answer is usually posed at an interaction point, and the instructor would typically discuss the given question in detail right after the interaction point. The findings above suggest that students seemed to be interested in accessing these questions.

However, although these questions usually draw students’ attention, some students may be satisfied once they obtained the correct answer to the question and they were less interested in exploring the concepts behind the question more. For example, in a video in which a spring-mass system is discussed, students were given the following clicker question in an interaction point: “*Suppose the period of a spring-mass oscillator is 1 s. What will be the period T if we double the spring stiffness? (We could use a stiffer spring, or we could attach the mass to two springs.)”* The number of unique students accessing specific in-video content dropped immediately after this interaction point by 13% (compared to the number of unique accessing students right before this interaction point.) We note that in the section of the video right after this interaction point, the instructor does not simply derive the answer *T* = 0.7 s from the equation $T=2\pi \sqrt{m/k}$ and then stop. Instead, he derives *T* = 0.7 s and then offers a sanity check for the result: *“the answer *T* = 0.7 s makes sense because period should get shorter when the spring becomes stiffer.”* This latter statement not only reinforces the concepts underlying the calculation of the spring’s oscillation period, but also demonstrates a good problemsolving strategy (the sanity check). While students who did not access this part of the video may still have been able to solve the clicker question correctly, they may not necessarily have mastered the concepts behind their answer nor performed their own sanity check. Detailed video interaction analysis can therefore point to possible directions for future instructor investigation, which may in turn help instructors design better ways to assist students in their learning.

## VI Discussion and Conclusions

In this study, student engagement of instructional videos in a flipped introductory mechanics course in which inclass lectures were replaced by online videos was explored. In this course, students were not graded on their video accesses. Our findings suggest that students may use the videos based on perceived value. For example, in this course, little in-class time was spent describing or working on laboratory activities. All instructions required for laboratory activities were delivered through laboratory videos. Students not only accessed the laboratory videos a lot, but also were more likely to access these videos completely without skipping sections in the laboratory videos when the video length increased. On the other hand, the fraction of students accessing lecture-oriented videos decreased to less than 40% toward the end of the semester. In addition, students were more likely to not access the lecture-oriented videos entirely when these videos become longer. Our finding that the fraction of students accessing the entirety of the video decreases with video length echoes the finding from a prior study [13] in which the relation between dropout rate and video length is explored.

However, in our study, an additional factor was taken into consideration by separating the videos into two groups based on different content types. It was found that the trend of students not accessing long videos completely was more prominent in the lecture-oriented videos than the laboratory videos. As suggested by student feedback on the end-of- course survey, the overall low access for lecture-oriented videos is likely due to factors such as students feeling that solving problems in class was sufficient (or more helpful) for learning the materials. The end-of-course survey also indicates that students tend to pay more attention to how to apply the physics concepts or principles learned, but the lecture videos did not provide as many examples as they would hope. This could be another reason why students are less motivated to access videos. Overall, our findings suggest that if an instructor wants to encourage students to access instructional videos, in addition to making videos short as suggested by the prior study, they should work to improve students’ perceived value of the videos (e.g., by explaining why the videos are important, discussing with students how they can best use these videos for learning, or directly pairing activities with relevant videos.)

In this study, we also found that there was little correlation between student engagement with instructional videos and their incoming background, and between student engagement with the videos and their performance in the course (see Table V). The latter result is similar to our past experiences from several semesters of traditional lecture-style introductory mechanics courses. However, with the advantage of having the lectures held online, we were able to explore in-depth how students interacted with different content of the lectures in an unprecedented way. Using the number of accesses and pauses as a proxy for students’ in-depth engagement with the video, the content that students focused on from two selected videos—the most accessed laboratory video and the most accessed lecture-oriented video—was explored. The results suggest that students seemed to engage more with concrete information that is explicitly required for assignment completion (e.g., actions required to complete laboratory work, or formulas or mathematical expression that are typically manifested explicitly in a problem solving task). However, students seemed to engage less with other types of content such as the underlying physical implication of a principle or the epistemology behind a principle.

To our knowledge, studies that explore how students interact with different content presented by instructors in a physics lecture are currently limited. In this study, we started our work on this issue by delving deeply into two example videos. Future work can extend this line of research to all videos in the course. We argue that these results can have great potential to help shape the instructional designs of the videos and/or the course structure to better support student learning. For example, we find that students appear to engage more with concrete information required to complete assignments than with explanations of concepts. This finding may be intertwined with the common result in physics education research that students may tend to focus on memorization and rote calculation than on sense making, and that students may solve quantitative problems correctly without necessarily mastering the underlying physics concepts. If a particular concept deemed important by the instructor was found to be less engaged by students from the clickstream data, an instructor can consider tailoring the in-class activities to reexamine students’ understanding of the given concept. If needed, the instructor can then help students contemplate the concept in more depth. In addition, our study also shows that students who accessed the interaction points did not necessarily access the instructor discussion that immediately follows. This suggests that the interaction points in the instructional videos can be designed with more thought in order for them to be more helpful. For example, if a clicker question is given at an interaction point, students may benefit from the clicker question more if all important concepts that an instructor wants to address with the given clicker question are explicitly reflected and incorporated in the question itself (as opposed to having some of them presented in the question and leaving the others to be addressed only in the discussion afterwards). It can also be helpful if the instructor encourages students to focus not only on the correctness of their answer to the clicker questions but also on the reasoning behind the answer more.

In addition to identifying the important content that may be less engaged by the students, an in-depth understanding of students’ video accessing behavior also has the potential of helping instructors address common student difficulties more effectively. For example, if students repeatedly access a concept presented in a video but continue to have great difficulties with that concept, instructors can reevaluate how the concept is presented in the video and prioritize that concept in future instruction. These results can also inform the instructors how to revise their videos to better fit their instructional goals.

In sum, the clickstream data can provide us with great insight into how students made use of online lectures in the course, which is a powerful and efficient tool that can help identify aspects of student learning suggestive for instructor interest for revision or future research. With iterative modification of the videos and/or course designs based on implications from these data, instructors can construct a more effective learning environment to better suit their instructional goals.

## Appendix: Methodology Details

### 1 Constructing matrix *A_ijkt_* from clickstream data

Three types of events (play, pause, and seek) are involved in the construction of matrix *A_ijkt_*. Remember that *A_tjkt_* represents how many times student *i* accessed the content at in-video time t of video *j* during the kth time she streamed this video. Therefore, for a given set of *i*, *j*, and *k*, every time the student clicked on the play button at in-video time *t*_1_ (in second) and then clicked on pause after the video proceeds to in-video time *t*_2_ (in seconds), a value of 1 is added to all elements between *A*_*ijkt*1_ and *A*_*ijkt*2_. Streaming sessions in which seek events are involved require more work. Students can, for example, watch the first 40 s of a video, and then skip to the point that corresponds to 100 s in in-video time, and then continue watching the video for another 20 s. Depending on whether the student had clicked on “pause” before she skipped to t = 100 s in the video, two patterns of recorded events are possible. The first type of pattern shown in Table VIII corresponds to the case in which students had clicked on pause before she skipped away from *t* = 40 s. The second type of pattern shown in Table IX corresponds to the case where students did not clicked on pause. We note that at the time when our course was offered, Coursera only recorded when a “seek” event ended, but not when it started. In the second case where a student skipped to a different point in the video while the video was still playing (i.e., the pause button had not been clicked), an estimation of the portion of video accessed before the seek event happened is required. This estimation is made by multiplying the difference in real time between the seek event and the previous event by the average playback rate [28] the student used. In the example shown in Table IX, it is estimated that the portion of video accessed between the 1st play event and the 2nd seek event is *t* = 0 to *t* = 0 + 40 × 1 = 40 s.

**Table IX:** Example made-up data explaining how events would be recorded in clickstream (the first 3 columns) and how matrix *A_ijkt_* would be constructed (the last 2 columns) for the following scenario: A student watched the first 40 seconds of a video, and then (without clicking on “pause” first) clicked on the scrubber below the video to jump to the point that corresponds to 100 s in in-video time. Since the pause button had not been clicked, the video would automatically continue playing from *t* = 100 s. The student then continued watching the video for another 20 seconds. In our study, we assume that the real-time it takes for the video to jump from invideo time *t* = 40 to *t* = 100 s is negligible. The playback rate student used was 1.

Event	Real time (s)	In-video time (s)	Delta Real time (s)	Implication for the elements in array A_ijk_	
Play	0	0	...	A 1 is added to every element	
Seek	40	100	40	*A_ijkt_* for *t* in [0; 0 + 40 × 1]	A 1 is added to every element
Pause	60	120	20	between *A*_*ijk*100~_*A*_*ijk*120_

### 2 Mathematical definition of a few variables useful for understanding students’ video accessing behaviors

(1) TS_*ij*_;: Total number of sessions (i.e., how many times student *i* has clicked on the link for video *j* to either access its content online or to download it)

$${\mathrm{TS}}_{ij}={\mathrm{TS}}_{ij\;\mathrm{from}\;\mathrm{streaming}}+{\mathrm{TS}}_{ij\;\mathrm{from}\;\mathrm{downloading}}=\left\{\begin{array}{ll} \max\left(k\right) & |\sum_{t}A_{ijkt}>0\\ 0 & \mathrm{if}\;\;\sum_{t}A_{ijkt}=0\;\mathrm{for}\;\mathrm{any}\;\mathrm{given}\;k \end{array}\right\}+D_{ij}.$$

(2) US_*ij*_: Unique (i.e., whether student i has ever clicked on the link for video *j* to access its content through online streaming or downloading

$${\mathrm{US}}_{ij}=\left\{\begin{array}{ll} 1 & \mathrm{if}\;{\mathrm{TS}}_{ij}\neq 0\\ 0 & \mathrm{if}\;{\mathrm{TS}}_{ij}=0 \end{array}\right..$$

(3) V_*i*_: Fraction of unique videos accessed by a student (i.e., what proportion of all videos in the course (*N_v_* = 78) student *i* has ever accessed)

$$V_{i}=\frac{\sum_{j=1}^{N\upsilon }\;{\mathrm{US}}_{ij}}{N\upsilon }.$$

When further separating the videos into lecture- oriented videos (*N*= 64) and laboratory videos (*N* = 14), similar fractions can be defined for videos with different content type:

$$V_{i,lec}=\frac{\sum_{j}\;{\mathrm{US}}_{ij\;\;}\mathrm{for}\;j\in \;\mathrm{Lecture}-\mathrm{oriented}\;\mathrm{videos}}{64}$$

$$V_{i,lab}=\frac{\sum_{j}\;{\mathrm{US}}_{ij\;\;}\mathrm{for}\;j\in \;\mathrm{Laboratory}\;\mathrm{videos}}{14}$$

(4) *C(V)*: Complementary cumulative distribution function of the amount of unique videos accessed by students (i.e., what fraction of students have accessed more than a certain proportion of videos in the course. Here, *V* represents the fraction of videos in a category). In particular,

$$C\left(V\right)=\frac{\mathrm{Card}\left(\left\{V_{i}|V_{i}>V\right\}\right)}{N_{s}},\;0\leq V\leq 1,$$

where Card() represents the cardinality, i.e., number of elements, of a given set.

When further separating the videos into lecture- oriented videos and laboratory videos, similar functions can be defined for videos with different content type:

$$C{\left(V\right)}_{lec}=\frac{\mathrm{Card}\left(\left\{V_{i,lec}|V_{i,lec}>V\right\}\right)}{N_{s}},\;0\leq V\leq 1,$$

$$C{\left(V\right)}_{lab}=\frac{\mathrm{Card}\left(\left\{V_{i,lab}|V_{i,lab}>V\right\}\right)}{N_{s}},\;0\leq V\leq 1,$$

(5) NS*_j_*: Number of students who has ever accessed a given video *j* through online streaming or downloading,

$${\mathrm{NS}}_{j}=\sum_{i=1}^{N_{s}}{\mathrm{US}}_{ij}.$$

(6) FS*_j_*: Fraction of students accessing a video (i.e., out of all the students in the course, what proportion of students have ever accessed the given video *j* through online streaming or downloading)

$${\mathrm{FS}}_{j}=\frac{{\mathrm{NS}}_{j}}{N_{s}}.$$

(7) TA_*ijt*_: Total number of accesses for specific in-video content by a single student (i.e., for a selected video *j*, how many times has student *i* accessed the content at t s of the given video). Because of the availability of data, this variable as well as the next three variables that follow are constructed only for cases of online video streaming. The few cases of video downloading [26] were excluded in such a type of analysis.

$${\mathrm{TA}}_{ijt}=\sum_{k=1}^{\max\left(k\right)}A_{ijkt}.$$

(8) UA_*ijt*_: Unique access for specific in-video content by a single student (i.e., for a selected video *j*, whether student , has ever accessed the content at ts of the given video),

$${\mathrm{UA}}_{ijt}=\left\{\begin{array}{ll} 1 & \mathrm{if}\;{\mathrm{TA}}_{ijt}\neq 0\\ 0 & \mathrm{if}\;{\mathrm{TA}}_{ijt}=0 \end{array}\right.,$$

(9) FC*_ij_*: Fraction of in-video content accessed (i.e., total fraction of video *j* that student , has ever accessed)

$${\mathrm{FC}}_{ij}=\frac{\sum_{t=1}^{t_{j}}\;{\mathrm{UA}}_{ijt}}{t_{j}}.$$

(10) FSE*_j_*: Fraction of students accessing “almost” the entirety of video *j* (i.e., out of all the students who accessed a selected video *j* through online streaming, what proportion of them have accessed “at least 99%” of the content in the given video.) FSE*_j_* is defined as

$${\mathrm{FSE}}_{j}=\frac{\mathrm{Card}\left(\left\{{\mathrm{FC}}_{ij}|FC_{ij}\geq 0.99,i=1\sim N_{s}\right\}\right)}{{\mathrm{NS}}_{j,\mathrm{streaming}}}$$

where NS_*j,streaming*_ represents the total number of students who has ever accessed video *j* through online streaming and is defined as

$${\mathrm{NS}}_{j,\mathrm{streaming}}=\sum_{i=1}^{N_{s}}{\mathrm{US}}_{ij,\mathrm{streaming}}$$

$$\mathrm{where}\;\;{\mathrm{US}}_{ij,\mathrm{streaming}}=\left\{\begin{array}{ll} 1 & \mathrm{if}\;\;{\mathrm{TS}}_{ij,\;\mathrm{from}\;\mathrm{streaming}}\neq 0\\ 0 & \mathrm{if}\;\;{\mathrm{TS}}_{ij,\;\mathrm{from}\;\mathrm{streaming}}=0 \end{array}\right..$$
